# Accuracy of Mobile Applications versus Wearable Devices in Long-Term Step Measurements

**DOI:** 10.3390/s20216293

**Published:** 2020-11-05

**Authors:** Filippo Piccinini, Giovanni Martinelli, Antonella Carbonaro

**Affiliations:** 1 Istituto Scientifico Romagnolo per lo Studio e la Cura dei Tumori (IRST) IRCCS, 47014 Meldola (FC), Italy; giovanni.martinelli@irst.emr.it; 2 Department of Computer Science and Engineering (DISI), University of Bologna, 47521 Cesena, Italy; antonella.carbonaro@unibo.it

**Keywords:** health and fitness datasets, step measurements, fitness trackers, wearable devices, mobile applications, long-term analysis

## Abstract

Fitness sensors and health systems are paving the way toward improving the quality of medical care by exploiting the benefits of new technology. For example, the great amount of patient-generated health data available today gives new opportunities to measure life parameters in real time and create a revolution in communication for professionals and patients. In this work, we concentrated on the basic parameter typically measured by fitness applications and devices—the number of steps taken daily. In particular, the main goal of this study was to compare the accuracy and precision of smartphone applications versus those of wearable devices to give users an idea about what can be expected regarding the relative difference in measurements achieved using different system typologies. In particular, the data obtained showed a difference of approximately 30%, proving that smartphone applications provide inaccurate measurements in long-term analysis, while wearable devices are precise and accurate. Accordingly, we challenge the reliability of previous studies reporting data collected with phone-based applications, and besides discussing the current limitations, we support the use of wearable devices for mHealth.

## 1. Introduction

Innovative applications (apps) and smart devices such as smartphones and tablets have emerged as integral parts of people’s lives [1]. They provide information to profile society [2], but also to make users aware of body signals important for predicting life expectancy [3], promoting patient engagement, self-management of diseases, and assist doctors to remotely follow up patients [4]. In particular, in the healthcare sector, we have seen the development of systems to assist users in different ways during the day [5] and night [6]. These devices offer incredible potential to generate big data, often identified as patient-generated health data (PGHD), that could influence medical doctors’ decision-making [7], as well as the potential for earlier diagnosis [8]. The World Health Organization (WHO) defines these tools under the labels electronic health (eHealth) and mobile health (mHealth): eHealth refers to any use of information and communication technology for health care; mHealth is a subset of eHealth specifically referring to the use of mobile and wireless devices. For instance, fitness trackers, blood glucose meters [9], blood pressure monitors [10], smoking sensors [11], and temperature-detection devices [12] are today popular for monitoring different fitness parameters and vital signs used for health analysis [13] and many other motion-related applications including tracking, positioning, activity recognition, and augmented reality [14]. In addition, electronic textiles (e-textiles), smart clothes, and flexible/printable electronics bring us closer to scenarios where electronic systems are totally integrated in our everyday life and help us in achieving a higher comfort level when interacting with smartphones and domotics in general [15].

Actually, there are three main ways to monitor and record physical activity (PA) parameters: (a) subjective methods, including self-reporting instruments such as questionnaires and diaries; (b) phone-based applications that help to record activities performed daily; and (c) wearable devices that record fitness and vital parameters in a continuous manner. However, most of the recent studies are based on mobile applications and/or fitness trackers to avoid subjective measurement, leading to wrong estimations (e.g., typically lower than self-reported estimations [16]). Among the fitness trackers, bracelets usually worn on the wrist can be today considered as the gold standard device used for activity tracking [17]. Furthermore, most of these fitness bracelets have complementary applications for interacting with social media to allow users to post their sleep and activity data, making these devices always more and more common. This has spawned a new trend, especially in the younger generations, and the total sales of fitness trackers are projected to be USD 6 billion by the end of 2020 [18].

Recently, Arigo et al. [19] described the history and future of the wearable devices used in medicine. Briefly, the earliest uses of technology to support medicine interventions occurred in the late 1940s and 1950s with the use of mechanical counters to provide feedback on users’ behavior. Pedometers (meant as early-stage mechanical devices that collect ambulatory data) were first used in the treatment of obesity in 1949 [20]. Later versions of activity sensors, such as aligned wrist- and ankle-worn counters [21], paved the way for modern smartphones and wearable fitness trackers. Most of these systems are equipped with accelerometers that record accelerations in one or more planes. These data elements are then processed into more meaningful variables [7], such as step counts; time spent in sedentary, light, moderate, and vigorous PA; flights of stairs climbed; and hours of sleep [22]. Steps are the basic parameter for several other indirect measurements. They are objective, intuitive, and comprehensible in the context of understanding personal activity, which makes this measure ideal for people to reflect on [23]. Steps are recorded in the device when a vertical acceleration deflects a spring-suspended lever arm above a designated force sensitivity threshold [24]. Most of the other common fitness tracker measures (e.g., flights of stairs, active minutes, calories burned, etc.) are then derived from the step count [25]. However, questions remain about the accuracy of the data they collect [26] and whether appropriate validation occurs before commercial sale [27].

In recent years, many studies have shown the importance of PA for long-term cardiovascular health [28]. Similarly, the number of studies considering patients’ PA in cancer clinical trials is rapidly increasing. Recently, Cox et al. [29] reviewed the different types of data recorded in cancer clinical trials, Purswani et al. [24] reviewed oncological studies specifically considering the patient’s steps, and Gresham et al. [30] looked at oncology trials based on wearable activity monitors. In general, the trend shows a rising number of studies involving some form of PA analysis, and the population is always more willing to share personal data, especially for research purposes [13]. In oncology, PGHD may be useful in providing continuous, dynamic, and objective assessments of patients’ health status between clinician visits. In general, medical doctors typically suggest that oncology patients engage in several PAs to keep a healthy status [31]. However, for several cancer types, the effects of PA on cancer prevention, treatment, and survival are still not known. An example is thyroid cancer for which the literature reports controversial results. Kitahara et al. [32] observed an increased risk of thyroid cancer in subjects reporting a higher frequency of PA. Conversely, the results reported by Leitzmann et al. [33] suggest that PA is unrelated to total thyroid cancer. Finally, Rossing et al. [34] support the hypothesis that PA may reduce the risk of thyroid cancer, but such a hypothesis is too sparse to make a more definitive judgment and give robust guidelines to patients. Like for thyroid, for several other cancer types we can also find in the literature controversial studies confirming that today, the health and fitness data at our disposal are not able to clarify the relations between cancer and PA. New technologies for collecting fitness and health data and better data accuracy may significantly improve the reliability of scientific studies in the field [35], and having large datasets reporting timeline PA and patients’ clinical status at the scientific community’s disposal would help in better profiling patients and understanding the correlations in order to improve people’s wellbeing [36].

**Figure 1:**
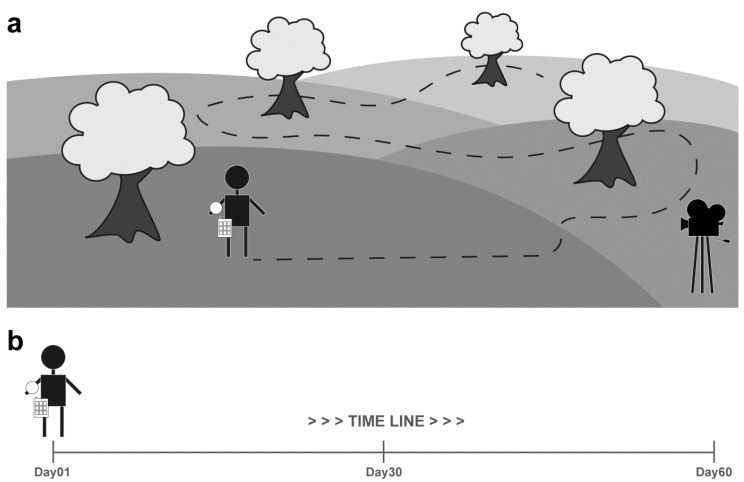
Representation of performed experiments. (**a**) Outdoor controlled test with number of steps counted by processing a video recorded with a camera. (**b**) Two-month 24 h/7 days monitoring of a healthy 35-year-old man wearing three fitness wristbands and carrying a mobile with six running step-counter applications.

In this work, we considered a basic parameter for PA evaluation—the number of steps taken daily. Our aim was to give the users an idea about how much steps they miss when measuring them through a smartphone-based application. In particular, we compared six fitness tracking applications for Android mobiles and three commercial fitness tracking wristbands, and we analyzed the precision and accuracy of the phone-based applications versus the dedicated wearable devices. Several brands and models of consumer trackers were examined for their accuracy in step measurement in a laboratory setting [37], e.g., treadmill walking [38], level walking [39], and stair walking [40]. However, count accuracy in real environments remains a major challenge [41]. To assess the accuracy and precision of the considered trackers, we performed two types of experiments (Figure 1). The first experiment was similar to those performed by Case et al. [42] and Modave et al. [43]. Basically, we evaluated the accuracy of the trackers in controlled outdoor tests with known ground truth, i.e., the real number of steps counted manually by analyzing videos recorded with a camera to serve as the benchmark (Figure 1a) [44]. In the second experiment, we asked a healthy 35-year-old man to install the six applications in his mobile and at the same time, wear all the fitness wristbands, and we measured the precision of the different systems in a 2-month 24 h/7 days experiment (Figure 1b). All the collected data and acquired videos are publicly available on FigShare (https://doi.org/10.6084/m9.figshare.c.4923645.v2).

The results obtained show that despite the good accuracy of all the tracking systems, the mobile applications did not provide reliable data in the long-term analysis due to an intrinsic problem, a missing parameter—the length of time the mobile was carried. Basically, in daily routines, mobiles are not always carried by people, especially when acting in a small indoor environment and performing standard daily life activities (e.g., cleaning, cooking, visiting the toilet), and this may generate unreliable day-based measurements. Generally, most of the time, young people carry their mobiles, so the number of steps recorded is reliable, but this is a very different situation for elders. In our case, even though the man performing the experiment was aware of the purpose of the collected data, the difference between the number of steps recorded by the phone-based applications was, on average, 30% lower with respect to what was recorded by the fitness wristbands, and this probably was misleading for all types of subsequent statistics, especially if the data were collected for clinical trials. Furthermore, these noncredible data may be understood by patients as being trustworthy and may also have a negative impact on users’ behavior.

We decided to share our data to make the scientific community aware that many studies based on steps counted with smartphone-based applications may be unreliable. Several recent as well as ongoing studies are based on data collected using smartphone-based applications [45], but our data show that the findings of these studies should be reconsidered. In our opinion, the only way to collect reliable data is to use an accurate fitness tracker worn on someone’s body for 24 h a day, 7 days a week. A major limitation of these devices is that they may not be sensitive enough for non-ambulatory physical activities such as cycling, swimming, and dancing [24]. Furthermore, most of them become unreliable when used for people with disabilities [46]. However, they are still a better compromise available today to collect reliable data passively without influencing people’s lives.

## 2. Materials and Methods

### 2.1. Phone-Based Applications and Wearable Fitness Trackers

Today, the market offers a wide range of phone-based applications and wearable devices for fitness tracking [47]. They differ in terms of available hardware and software installed, and it is known that the accuracy depends on both software and hardware [48]. However, even when the operating system, application programming interface (API), and sensors are the same, different implementations can affect the computation of the number of steps.

In this work, we mimicked a user interested in installing an application for fitness tracking by downloading one from among those freely available. The applications and devices included in this study were selected considering different software companies, prices, sensors, and algorithms used to analyze the data. Our decision was to install the applications in a mid-range smartphone, considering this to be the representative technology of the average consumer today. In particular, all of the applications were downloaded from the Google Play store and were installed on a Huawei P Smart FIG-LX1 phone with the Android 9 operating system.

In this work, 6 phone-based fitness tracking applications (APPs) and 3 wearable fitness trackers (WFTs) were tested. The 6 applications were:

- (APP1) Huawei Health, v10.0.2.333 (https://consumer.huawei.com);
- (APP2) Bits&Coffee ActivityTracker, v1.2.2.400070 (https://activitytrackerapp.com);
- (APP3) Best Simple Apps Contapassi, v4.1.5 (smartandusefulapps@gmail.com);
- (APP4) GALA MIX WinWalk, v1.9.6 (http://winwalk.club);
- (APP5) LG Electronics LG Health, v5.40.16 (https://www.lg.com);
- (APP6) Pacer Health’s Pacer, vp6.10.1 (https://www.mypacer.com).

The 3 wearable fitness trackers were:

- (WFT1) Decathlon OnCoach 100, v1.1.6(39), price ~EUR 25 (https://www.decathlon.com);
- (WFT2) Crane activity tracker, v1.45, price ~EUR 50 (https://www.suunto.com);
- (WFT3) Suunto 9, v4.17.4, price ~EUR 500 (https://consumer.huawei.com).

### 2.2. Tracker Accuracy: Experiment Description

To assess how closely the different trackers agreed, we asked 3 operators to perform a test carrying a mobile with the 6 apps installed in a trouser pocket and wearing the 3 fitness wristbands on their left arm. The operators were 3 healthy subjects: a 35-year-old man (hereafter, Operator1), a 65-year-old man (Operator2), and a 65-year-old woman (Operator3). We asked each operator to walk in a fairly flat park while counting approximately 1200 steps. For the test, we chose this outdoor environment and not an indoor treadmill because it is known that in real-world conditions, especially on difficult terrain, there can be far more variation in step counts, given the changes in gait and wrist movement [43]. To have the ground truth, a cameraman recorded a video by following the operator performing the test with a camera. The acquired videos were post-processed to count the actual number of steps. Accuracy was then assessed by comparing the number of steps recorded by each tracker with the ground truth. All videos considered in the experiments are publicly available on FigShare (https://doi.org/10.6084/m9.figshare.c.4923645.v2).

### 2.3. Tracker Precision: Experiment Description

To evaluate the precision of the different trackers, we performed a long-term experiment based on the one-day test lasting for a few months in order to collect 60 days of reliable data. Basically, we asked an operator to carry the mobile with apps installed and wear the fitness wristbands 24 h/7 days. Then, we kept the data of the first 60 days, in which all the tracking systems were working. Precisely, every time one of the tracking systems was under recharge, we discarded the data collected by all the other systems. Furthermore, all the days when the operator could wear the wristbands but not carry the mobile in a trouser pocket (due to performing activities in his free time that could potentially damage the smartphone, for instance swimming, working in a vegetable garden, riding a horse, dancing) were removed from the analysis.

The operator was Operator1, a healthy 35-year-old man who leads a standard office job life (he is a computer scientist researcher) with several typical activities like working at the computer, answering the office phone, going to the printer, sharing documents with colleagues, and meeting with collaborators. The raw data recorded by different trackers are reported in Table 1. Once again, it is worth remarking that the 60 days considered in the experiment were not continuous, mainly due to battery recharge issues of the wearable devices and operator needs.

### 2.4. Statistics

In the accuracy assessment, the absolute normalized difference percentage (PAND) between step value (*V_i_*) recorded by the trackers (*i*) and ground truth (*G*), determined in the post-processing of the videos, was computed to measure the closeness of agreement of the different counts according to Equation (1):

PAND = 100 × |*V_i_* − *G*|/*G* (1)

In the precision assessment, unbalanced one-way analysis of variance (ANOVA) was performed to measure if the step values recorded daily by the apps significantly differed from those recorded by the fitness wristbands. Then, the normalized difference percentage (PND) between the values (*V_i_*) recorded by the trackers (*i*) and the average value (*A*), computed considering the values of apps (*A_a_*) or wearables (*A_w_*) together, was calculated to evaluate the precision of each tracker *i*, according to Equation (2):

PND = 100 × (*V_i_* − *A_a/w_*)/*A_a/w_* (2)

The tests were carried out using MATLAB (MathWorks, Inc., Natick, MA, USA), and a *p*-value ≤ 0.05 was considered statistically significant. The asterisks in column 1 of Table 2 are only intended to flag levels of significance for three of the most commonly used levels: *p*-value less than 0.05 is flagged with one asterisk (*), *p*-value less than 0.01 is flagged with two asterisks (**), and *p*-value less than 0.001 is flagged with three asterisks (***). The bold values reported in column 1 of Table 2 highlight the days when the step values recorded by the apps did not significantly differ (considering *p*-value = 0.05 as the threshold) from those recorded by the fitness wristbands. The same analysis was repeated by accumulating the steps and subdividing the data in 6 blocks of 10 days (Table 3).

## 3. Results

### 3.1. Tracker: Accuracy: Results

**Figure 2:**
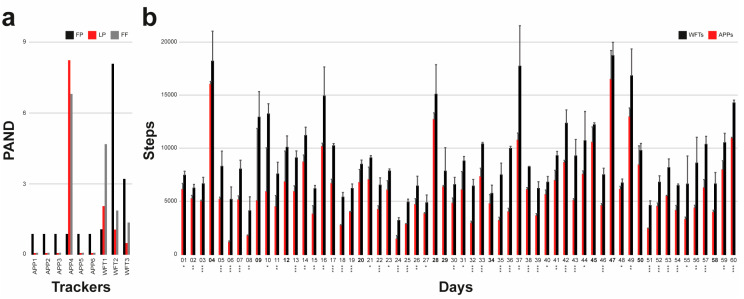
Bar-charts showing (**a**) absolute normalized difference percentage (PAND) values reported in the last nine rows of Table 1, and (**b**) day-based averages of the APPs and WFTs step values reported in Table 2. The gray bars in (**b**) are the standard deviations. * *p*-value less than 0.05; ** *p*-value less than 0.01; *** *p*-value less than 0.001. Bold values in the *x*-axis of (**b**) highlight days when step values recorded by APPs do not significantly differ (considering *p*-value = 0.05 as the threshold) from those recorded by fitness wristbands (WFTs).

To assess the accuracy of the trackers, we asked three operators to wear the devices and walk in a park while counting approximately 1200 steps. To have the ground truth number of steps, a cameraman followed the operator and recorded a video that was subsequently used to determine the real number of steps. Table 1 reports the name of the operator performing the test in row 1, the ground truth number of steps in row 2, the number of steps recorded by the devices in rows 3–11, and the PAND values in rows 12–21. It is worth noting that all apps counted the same number of steps in all tests, except APP4 in the test performed by Operator2 and Operator3 (Figure 2a). However, all PAND values were lower than 10% (the worst PAND was 8.23% for the apps and 8.08% for the wristbands), proving the accuracy of all the trackers in a short-term analysis performed under the controlled conditions of a standard walk in a park.

### 3.2. Tracker Precision: Results

To analyze the precision of the trackers, PND values were computed considering *A_a_* as a normalization factor for apps and *A_w_* for the fitness trackers. Table 2 reports the steps counted by the trackers in the 60-day experiment. Day-based average values and standard deviations of APPs and WFTs are reported in Figure 2b. PND values are reported in Table 4. The average PND computed by considering all the data collected (last line of Table 4) ranges from −3% to 8% for apps and −6% to 4% for wearables, showing good precision of the trackers. The same analysis was repeated not just day-based, but also accumulating the steps and subdividing the data in 6 blocks of 10 days. Table 3 reports the cumulative step numbers and Table 5 the block-based PND values. The average PND computed by considering the 6 blocks together (last line of Table 5) ranges from −4% to 8% for apps and −8% to 4% for wearables. This shows that despite some local values strongly differing from the average, across the whole experiment, there were no significant differences between the APPs or WFTs considered separately. Accordingly, this proves that today, despite recorded values being logically dependent on the quality of the algorithms, on average, thanks to the available technology, all apps and wearables gave practically the same number of counted steps in long-term analysis.

However, the number of steps recorded by the phone-based apps was significantly lower than that recorded by the fitness wristbands. The average number of steps recorded daily by the phone-based apps was on average 34% lower than that recorded by the fitness wristbands (Figure 2b) according to the day-based analysis, and 32% in the cumulative-based one. Furthermore, on approximately 80% of the days (50 out of 60 days), the values recorded by the phone-based apps statistically significantly differed from those recorded by the wearable devices, according to an unbalanced one-way ANOVA test performed on the day-based data. This suggests lower accuracy of the apps in long-term analysis with respect to wearables worn on the body. This is also emphasized when considering the cumulative-based data reported in Table 3: in 100% of the 6 blocks, the cumulative values referring to the phone-based apps strongly (*Block I* is characterized by a *p*-value lower than 0.01; all the other blocks by *p*-values lower than 0.001) statistically significantly differed from those referring to the wearable devices.

It is worth remarking that the operator performing the experiment was well informed about the finality of the acquired data. However, to collect reliable data, he did not modify his daily routine and the way he used his mobile. Accordingly, he was not carrying the mobile during several daily movements typical of a standard office job, like standing from the desk for answering the office phone, going to the printer, bringing documents to colleagues, and visiting the toilet. Furthermore, during his free time (mainly in the evenings at home), he was not carrying the mobile during standard activities like cooking and cleaning. The recorded data proved that these standard movements strongly affect the final count of steps taken, and the difference between steps recorded by phone-based apps and fitness wristbands would be even larger for elderly people, who typically leave their mobile on a desk in a central room of the house and perform standard activities in the environment.

## 4. Discussion

One of the goals of this work was to compare the accuracy and precision of smartphone-based apps versus those of wearable fitness wristbands to suggest the best way to collect data for long-term clinical trials. Even though several research studies have confirmed that both the accuracy and precision of activity tracking devices have steadily improved over the last decade, device accuracy still remains a concern. However, technological improvements, increased knowledge on how to use the devices and for what purposes, and a better statistical approach to understanding the data all contribute to more comfort in the use of wearables and sensors for clinical trials.

Logically, to be effective in measuring PA, patients must wear a wristband or use an app for the entire day. Therefore, design, battery life, water-resistance, and comfort must all be maximized [29]. However, since in daily life people are not always carrying their smartphone, data collected by phone-based apps may be unreliable for long-term analysis, and asking people to carry their smartphone 24 h a day, 7 days a week would be impossible and would definitively change their lifestyle. Besides, other studies already proved that even fitness wristbands generally slightly underestimate the number of steps [38]. Consequently, there are several reasons to suggest that data obtained by smartphone-based apps are unreliable. On the other hand, fitness wristbands: (a) are somewhat invasive; (b) do not influence people’s lifestyle; and (c) can collect accurate and precise data for long-term analysis. However, since foot pods measure walking or running cadence directly, we do not exclude the idea that more reliable data could be acquired using ankle bracelets, which are probably more accurate than devices measuring at the wrist or the hip [43]. Previous comparisons of data from various consumer- and commercial-grade wearables already demonstrated variability among devices related to body placement. For instance, Hildebrand et al. [49] examined the effects of tracker placement on acceleration values. In particular, they showed a significant difference between hip and wrist placement, showing that the output from the wrist was generally higher than that from the hip. A hip tracker usually has several limitations, including underestimation of energy expenditure during activities with little or no movement at the hip and potential loss of data due to removing the tracker when dressing, and influencing the lifestyle. On the other hand, several studies have documented noncompliance resulting in loss of data when using a hip-mounted accelerometer [16,50].

Actually, how to improve the general accuracy of step counting still remains a very important issue. For instance: (a) by exploiting the decreasing trend of the prices of electronics and the increasing size-reduction of all the sensors, three sensors could be embedded in all the devices to always provide a median value; (b) GPS measurements of the distances that have been walked or ran could be considered in all step counting algorithms to improve the accuracy of the system; (c) other less noise-prone body placement positions could be tested, for instance the neck or waist, or smart wearable technologies, such as smart shirts or pants, could be considered. However, with the information available today, we think that the best trade-off in collecting reliable data without affecting people’s lifestyle is offered by wearable fitness wristbands. Therefore, we suggest that these types of devices be used for long-term clinical trials, and we caution the community to reconsider the findings of previous studies based on smartphone applications.

## 5. Conclusions

At this time, the number of clinical trials involving physical activity measurements is growing. Smartphone-based applications, fitness wristbands, ankle bracelets, hip belts, and many other tracking systems are very common in society. However, the accuracy and precision of these devices still remain a concern.

In this work, we analyzed the precision and accuracy of six phone-based applications (i.e., APP1—Huawei Health; APP2—Bits&Coffee ActivityTracker; APP3—Best Simple Apps Contapassi; APP4—GALA MIX WinWalk; APP5—LG Electronics LG Health; APP6—Pacer Health’s Pacer) and three wearable fitness wristbands (i.e., WFT1—Decathlon OnCoach 100; WFT2—Crane activity tracker; WFT3—Suunto 9). The experiments performed were conceived as a proof of concept to give an idea about what can be expected in terms of relative differences in the measurements achieved using different system typologies. In particular, the number of steps recorded by the trackers in controlled tests with ground truth was considered to assess the accuracy of the trackers; a long-term analysis based on data acquired in a 2-month experiment was considered to estimate the precision. The first experiment performed proves that the accuracy of the smartphone applications is comparable to that of the wearable fitness wristbands; the second experiment proves that due to the fact that people cannot always carry a smartphone, the step measurement in long-term analysis may differ even by 30%. Providing significant statistics about the absolute performance of mobile applications and fitness wristbands is beyond the scope of this work. However, we can reassume that between the applications, just APP4 gave significantly different results, and between the wristbands, WFT1 gave, in general, lower step measurements.

The main outcome of the experiments is that despite good accuracy in short-term analysis of controlled conditions, data acquired with phone-based applications may be unreliable in long-term analysis. This not due to system accuracy, but because people do not always carry their smartphone in their trouser pocket. For instance, they typically leave their mobile on a desk in a central room of the house and do not carry their smartphone when performing standard daily life activities (e.g., cleaning, cooking, visiting the toilet) in small indoor environments, and we proved that the sum of these moments, added to the moments when they do not carry their mobile because performing activities that could potentially damage it (e.g., swimming, working in the vegetable garden, riding a horse, dancing), at the end of the day, may have a significant impact on step measurement.

To conclude, besides suggesting the use of wearable fitness wristbands to collect data, we also caution the scientific community to reconsider outcomes of studies based on data collected with mobile trackers.

## Figures and Tables

**Table 1:** Steps recorded by trackers in step-controlled experiments.

Operator	Operator1	Operator2	Operator3
GROUND TRUTH [steps]	1027	1409	1175
APP1 [steps]	1036	1410	1176
APP2 [steps]	1036	1410	1176
APP3 [steps]	1036	1410	1176
APP4 [steps]	1036	1293	1095
APP5 [steps]	1036	1410	1176
APP6 [steps]	1036	1410	1176
WFT1 [steps]	1016	1380	1230
WFT1 [steps]	944	1424	1197
WFT1 [steps]	994	1402	1159
APP1 (PAND)	0.88	0.07	0.09
APP2 (PAND)	0.88	0.07	0.09
APP3 (PAND)	0.88	0.07	0.09
APP4 (PAND)	0.88	8.23	6.81
APP5 (PAND)	0.88	0.07	0.09
APP6 (PAND)	0.88	0.07	0.09
WFT1 (PAND)	1.07	2.06	4.68
WFT1 (PAND)	8.08	1.06	1.87
WFT1 (PAND)	3.21	0.50	1.36

**Table 2:** Steps recorded by trackers in the 2-month experiment. * *p*-value less than 0.05; ** *p*-value less than 0.01; *** *p*-value less than 0.001. Bold values in column 1 highlight days when step values recorded by APPs do not significantly differ (considering *p*-value = 0.05 as the threshold) from those recorded by fitness wristbands (WFTs).

Day	APP1	APP2	APP3	APP4	APP5	APP6	WFT1	WFT2	WFT3
01 *	6767	5000	6203	6100	6328	6140	7139	7216	7899
02 **	5507	5389	5070	5400	5310	4594	5907	6095	6631
03 ***	5015	5015	4760	5000	5082	5015	6114	6387	7326
04	16,234	16,150	15,469	16,100	15,951	16,150	15,082	20,687	18,774
05 ***	5363	5286	4823	4900	5233	5286	6596	9336	8853
06 ***	1188	1188	1004	1200	792	1188	4011	6405	5018
07 ***	5296	5255	4319	5300	5255	5255	7110	8817	8102
08 **	1793	1793	1589	1700	1793	1733	2759	3991	5452
09	13,997	629	13,699	629	629	629	10,112	13,966	14,618
10 *	11,305	3662	11,047	2000	3663	3674	12,090	14,023	13,480
11 **	5568	4337	4651	2600	4169	5443	8661	7564	6381
12	8846	2396	8426	3800	8708	8738	8835	10,962	10,372
13 ***	6299	6199	5959	4800	6101	6059	9633	8314	9241
14 **	9158	8931	7976	8000	8488	9621	10,356	11,972	11,192
15 **	4966	2764	4508	3400	3383	3591	5848	6504	6120
16 **	10,179	10,493	9640	10,000	10,493	10,103	11,770	17,065	15,826
17 ***	6926	6926	6046	6200	6924	6926	9917	10,366	10,291
18 ***	2777	2788	2631	2600	2604	2788	5159	5013	5893
19 ***	3948	3948	3846	4100	3907	3948	6666	5785	6068
20	6050	6154	9211	6900	6154	6048	8000	8831	8543
21 *	7798	7798	6052	5000	7652	7798	9306	8831	9062
22 ***	4036	3988	4858	4400	3988	3988	6354	5841	7265
23 *	6403	6403	6429	4200	6403	6403	7613	8051	7811
24 ***	1306	1306	1155	2100	1306	1306	3105	2880	3466
25 ***	2891	2862	2840	2700	2772	2862	4858	4592	5199
26 **	4741	4413	4259	5800	4399	4393	7262	6573	5391
27 *	3800	3800	3615	4000	3800	3800	5618	4752	4107
28	12,487	12,410	12,414	14,000	12,410	12,410	11,813	16,608	16,767
29	6442	6395	6107	6500	6252	6309	5275	9551	8598
30 **	5220	4987	4818	3800	4987	4987	6131	6130	7361
31 *	5505	5373	5154	9600	5370	5373	9108	8936	8252
32 ***	3067	3059	2740	2600	3057	2991	6646	5655	6898
33 ***	6972	7189	6748	8900	7187	6817	10,567	10,244	10,292
34	4645	4428	4015	6500	4428	4428	4838	5860	6421
35 ***	3188	3023	2967	3800	2964	3023	6174	7824	8364
36 ***	4045	3921	3565	4600	3921	3921	9744	10,198	9900
37 **	11,294	11,035	10,648	9400	11,035	11,035	13,264	19,936	19,899
38 ***	6323	6187	5705	6000	6041	6187	8226	8135	8289
39 ***	3919	3697	3278	3500	3563	3697	6939	5700	5900
40 *	6113	5809	5296	4900	5809	5809	7062	6118	7123
41 **	6765	6574	6232	8900	6574	6574	9763	9020	9004
42 ***	8707	8617	8305	9000	8587	8617	10,972	12,499	13,511
43 ***	5132	5129	5142	4700	5129	5129	7411	10,038	10,260
44 *	7439	7487	7252	8200	7311	7436	7536	11,673	12,831
45	11,226	11,226	10,800	7600	11,226	11,226	11,935	12,368	12,228
46 ***	4729	4731	4458	4200	4580	4729	6836	7442	8118
47	17,699	17,699	17,271	10,900	17,560	17,699	17,440	20,016	18,621
48 *	6055	6055	5863	6700	5851	6055	6396	6533	7151
49 **	13,563	13,188	13,309	11,200	13,188	13,188	13,889	17,786	18,717
50	7744	7744	7600	12,100	7606	7606	10,101	8938	10,198
51 ***	2473	2473	2400	2400	2407	2407	5109	4265	4340
52 ***	4694	4694	4631	3900	4498	4694	7346	6078	6859
53 ***	5578	5408	5456	5400	5385	5577	7974	7373	9056
54 ***	4435	4363	3986	3200	4363	4363	6572	6236	6530
55 *	3257	3228	2992	3700	3228	3228	4890	9674	5193
56 **	4279	4249	4120	4900	4246	4249	7162	11,407	7133
57 ***	6719	6553	6468	4600	6553	6553	9663	10,120	11,205
58	4304	3981	3807	3700	3917	3981	5891	6018	7882
59 **	7811	7651	7370	9700	7651	7532	9423	10,990	11,043
60 ***	10,967	10,967	10,752	11,000	10,967	10,967	14,003	14,171	14,559

**Table 3:** Cumulative number of steps recorded subdividing the data reported in Table 2 in 6 blocks of 10 days. Asterisks in column 1 highlight days when step values recorded by APPs significantly differ from those recorded by fitness wristbands (WFTs). * *p*-value less than 0.05; ** *p*-value less than 0.01; *** *p*-value less than 0.001.

Block	APP1	APP2	APP3	APP4	APP5	APP6	WFT1	WFT2	WFT3
I **	72,465	49,367	67,983	48,329	50,036	49,664	76,920	96,923	96,153
II ***	64,717	54,936	62,894	52,400	60,931	63,265	84,845	92,376	89,927
III ***	55,124	54,362	52,547	52,500	53,969	54,256	67,335	73,809	75,027
IV ***	55,071	53,721	50,116	59,800	53,375	53,281	82,568	88,606	91,338
V ***	89,059	88,450	86,232	83,500	87,612	88,259	10,2279	11,6313	12,0639
VI ***	54,517	53,567	51,982	52,500	53,215	53,551	78,033	86,332	83,800

**Table 4:** Percentage normalized difference (PND) values computed for trackers in the 2-month experiment.

DAY	APP1	APP2	APP3	APP4	APP5	APP6	WFT1	WFT2	WFT3
01	11	−18	2	0	4	1	−4	−3	6
02	6	3	−3	4	2	−12	−5	−2	7
03	1	1	−4	0	2	1	−7	−3	11
04	1	1	−3	1	0	1	−17	14	3
05	4	3	−6	−5	2	3	−20	13	7
06	9	9	−8	10	−28	9	−22	24	−2
07	4	3	−16	4	3	3	−11	10	1
08	3	3	−8	−2	3	0	−32	−2	34
09	178	−88	172	−88	−88	−88	−22	8	13
10	92	−38	87	−66	−38	−38	−8	6	2
11	25	−3	4	−42	−7	22	15	0	−15
12	30	−65	24	−44	28	28	−12	9	3
13	7	5	1	−19	3	3	6	−8	2
14	5	3	−8	−8	−2	11	−7	7	0
15	32	−27	20	−10	−10	−5	−5	6	−1
16	0	3	−5	−1	3	0	−21	15	6
17	4	4	−9	−7	4	4	−3	2	1
18	3	3	−2	−4	−3	3	−4	−6	10
19	0	0	−3	4	−1	0	8	−6	−2
20	−10	−9	36	2	−9	−10	−5	4	1
21	11	11	−14	−29	9	11	3	−3	0
22	−4	−5	15	5	−5	−5	−2	−10	12
23	6	6	6	−30	6	6	−3	3	0
24	−8	−8	−18	49	−8	−8	−1	−9	10
25	2	1	1	−4	−2	1	−1	−6	6
26	2	−5	−9	24	−6	−6	13	3	−16
27	0	0	−5	5	0	0	16	−2	−15
28	−2	−2	−2	10	−2	−2	−22	10	11
29	2	1	−4	3	−1	0	−32	22	10
30	9	4	0	−21	4	4	−6	−6	13
31	−9	−11	−15	58	−11	−11	4	2	−6
32	5	5	−6	−11	5	2	4	−12	8
33	−5	−2	−8	22	−2	−7	2	−1	−1
34	−2	−7	−15	37	−7	−7	−15	3	13
35	1	−4	−6	20	−6	−4	−17	5	12
36	1	−2	−11	15	−2	−2	−2	3	0
37	5	3	−1	−12	3	3	−25	13	12
38	4	2	−6	−1	−1	2	0	−1	1
39	9	2	−9	−3	−1	2	12	−8	−5
40	9	3	−6	−13	3	3	4	−10	5
41	−2	−5	−10	28	−5	−5	5	−3	−3
42	1	0	−4	4	−1	0	−11	1	10
43	1	1	2	−7	1	1	−20	9	11
44	−1	0	−4	9	−3	−1	−29	9	20
45	6	6	2	−28	6	6	−2	2	0
46	3	3	−2	−8	0	3	−8	0	9
47	7	7	5	−34	7	7	−7	7	0
48	−1	−1	−4	10	−4	−1	−4	−2	7
49	5	2	3	−13	2	2	−17	6	11
50	−8	−8	−10	44	−9	−9	4	−8	5
51	2	2	−1	−1	−1	−1	12	−7	−5
52	4	4	2	−14	0	4	9	−10	1
53	2	−1	0	−1	−2	2	−2	−9	11
54	8	6	−3	−22	6	6	2	−3	1
55	0	−1	−9	13	−1	−1	−26	47	−21
56	−1	−2	−5	13	−2	−2	−16	33	−17
57	8	5	4	−26	5	5	−6	−2	8
58	9	1	−4	−6	−1	1	−11	−9	19
59	−2	−4	−7	22	−4	−5	−10	5	5
60	0	0	−2	1	0	0	−2	−1	2
AVERAGE	8	−3	2	−3	−3	−1	−6	2	4

**Table 5:** Percentage normalized difference (PND) values computed analyzing the 6 blocks of cumulative steps reported in Table 3.

Block	APP1	APP2	APP3	APP4	APP5	APP6	WFT1	WFT2	WFT3
I	29	−12	21	−14	−11	−12	−15	8	7
II	8	−8	5	−12	2	6	−5	4	1
III	2	1	−2	−2	0	1	−7	2	4
IV	2	−1	−8	10	−2	−2	−6	1	4
V	2	1	−1	−4	0	1	−10	3	7
VI	2	1	−2	−1	0	1	−6	4	1
AVERAGE	8	−3	2	−4	−2	−1	−8	4	4
